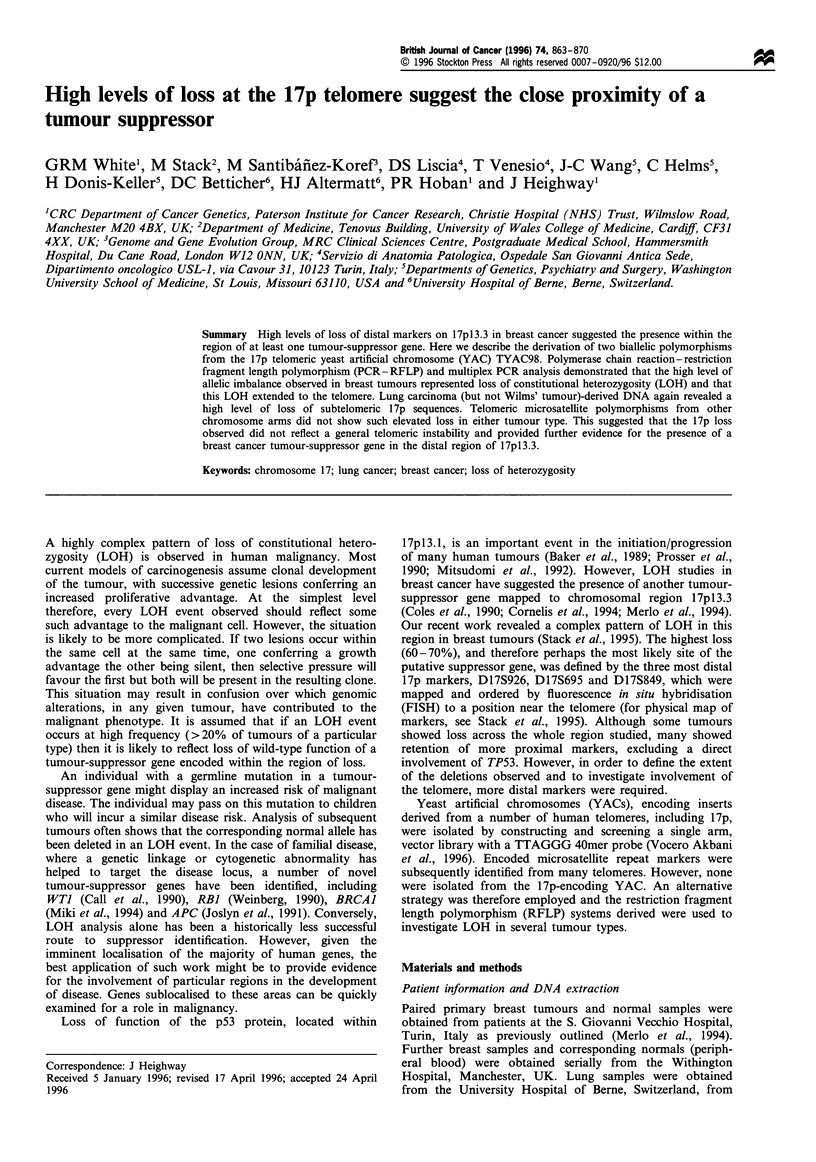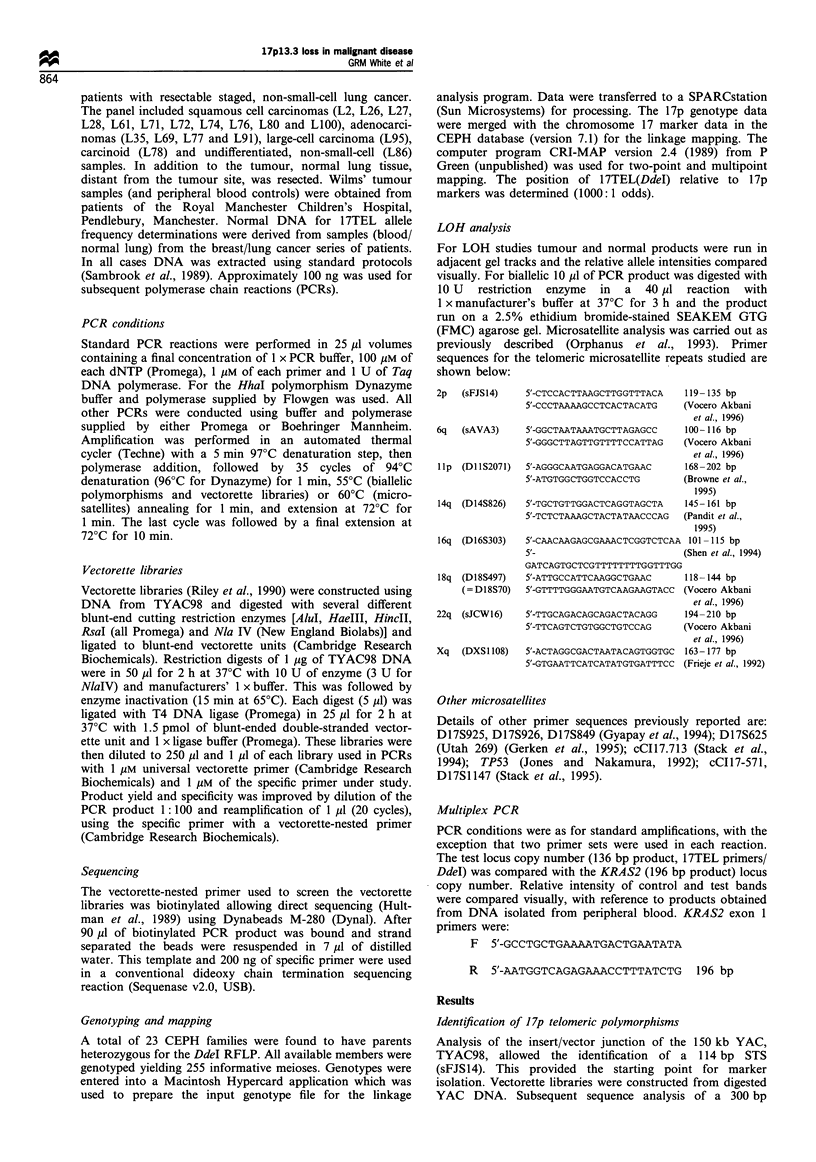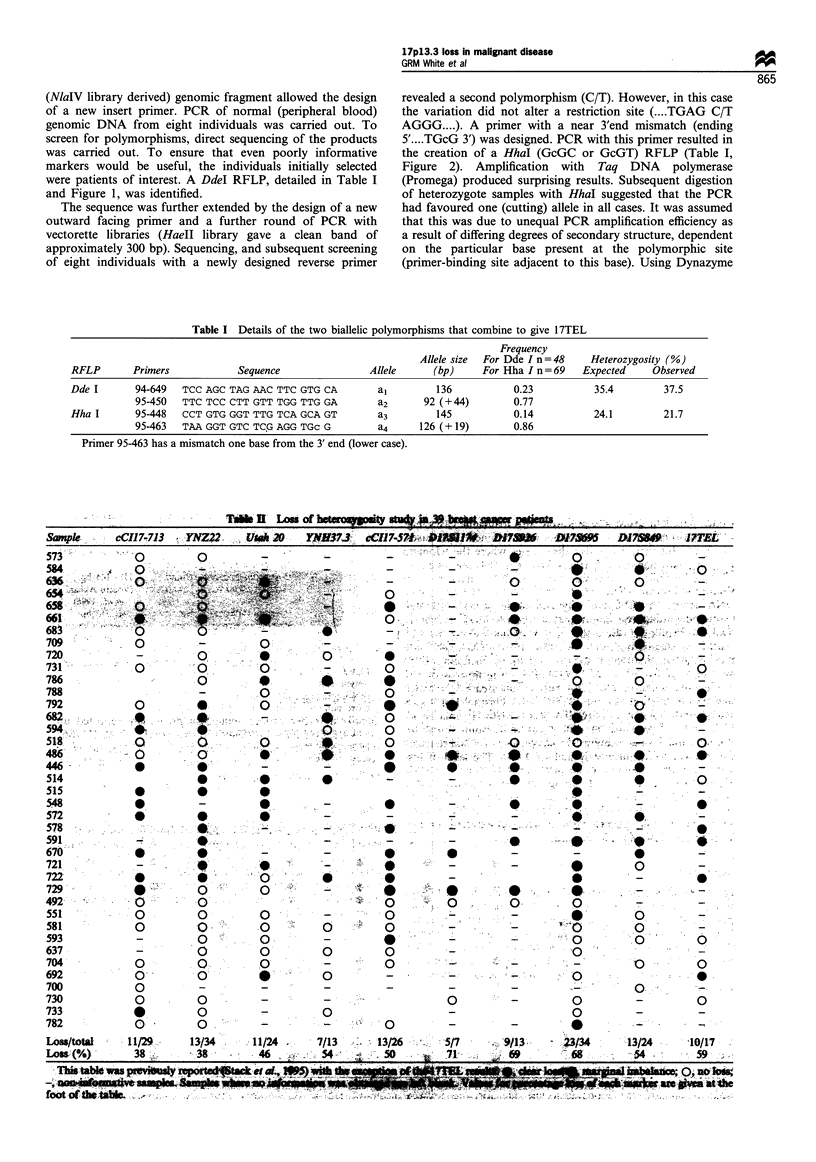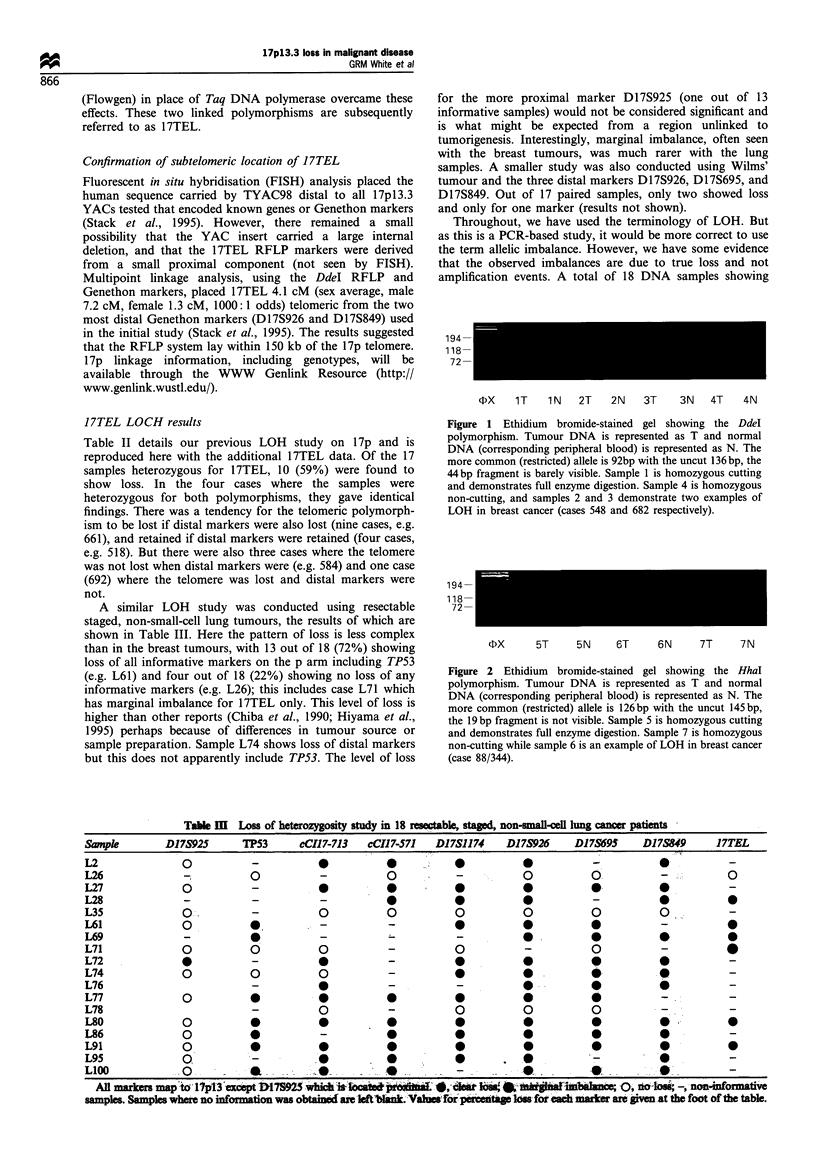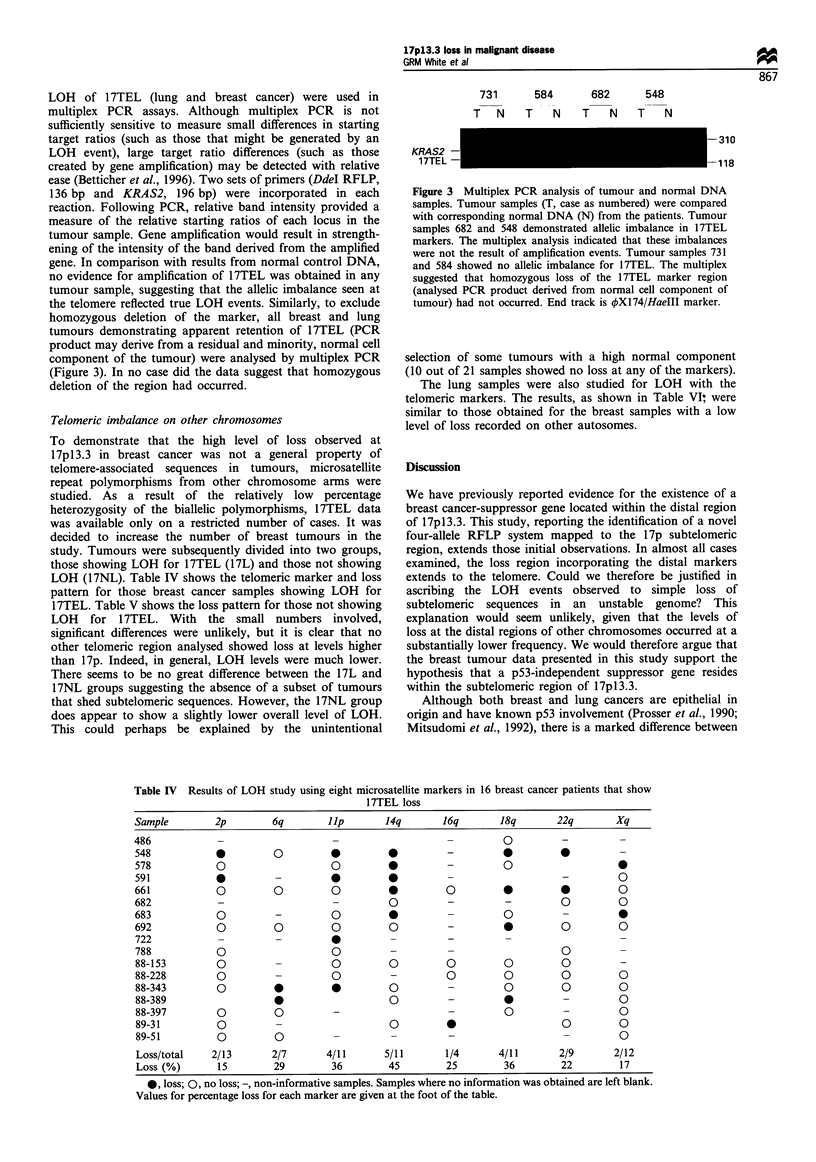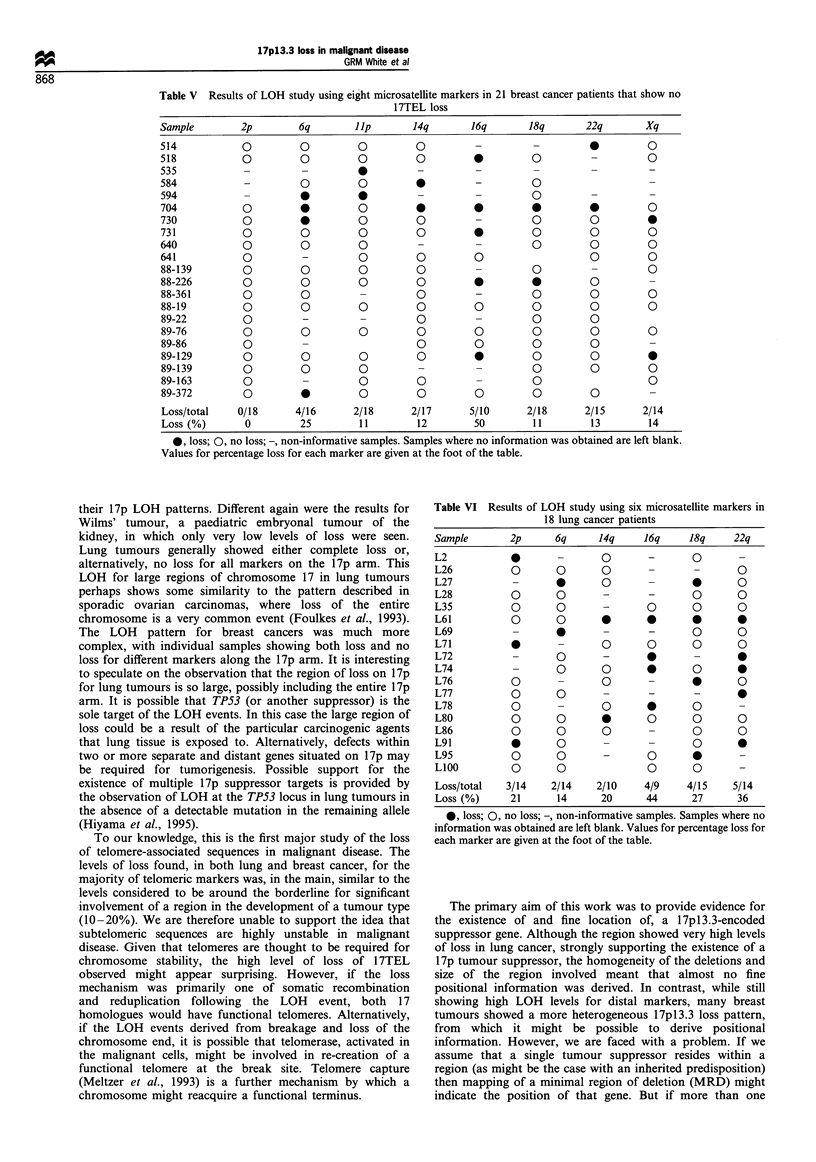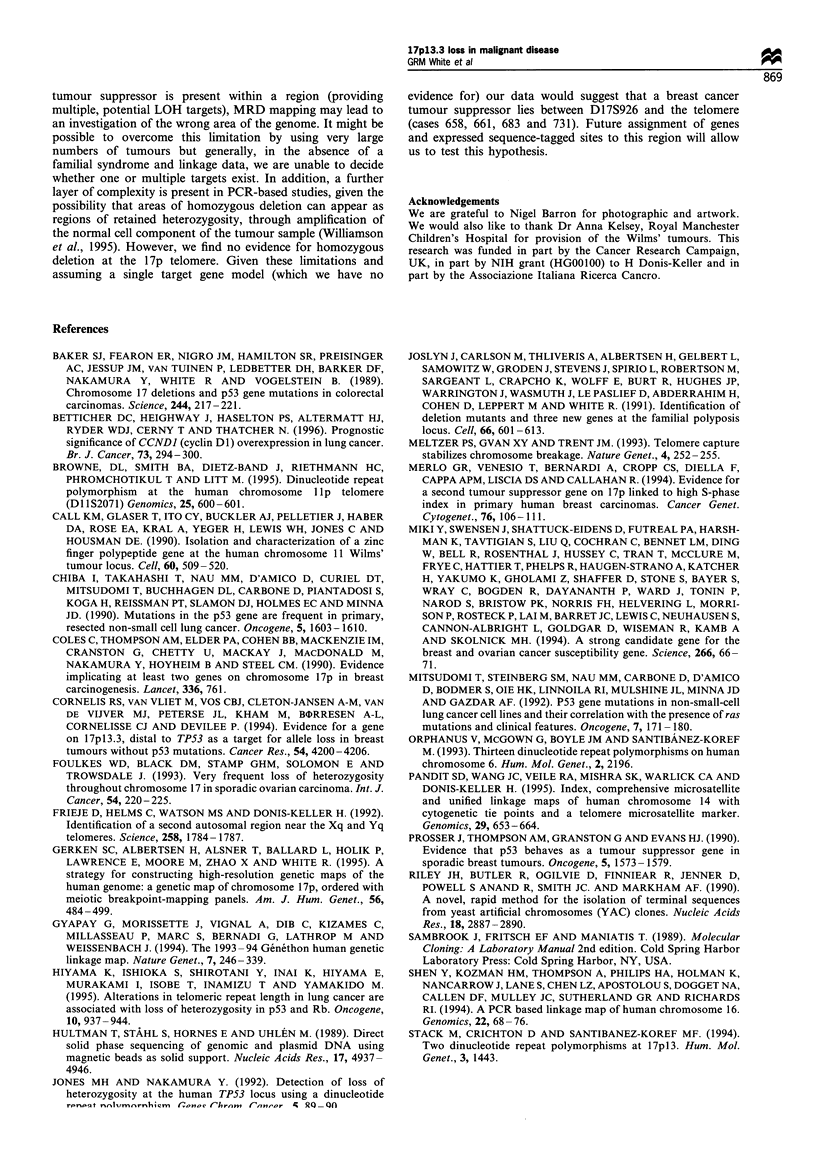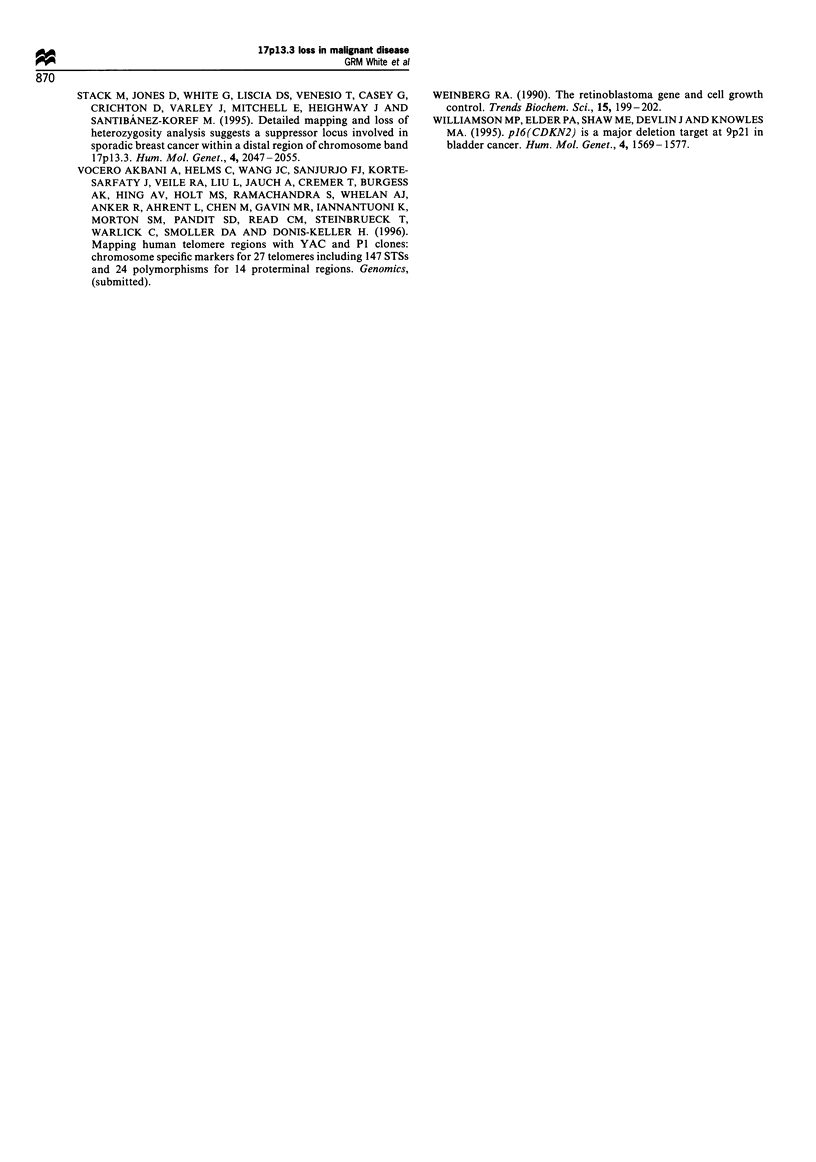# High levels of loss at the 17p telomere suggest the close proximity of a tumour suppressor.

**DOI:** 10.1038/bjc.1996.449

**Published:** 1996-09

**Authors:** G. R. White, M. Stack, M. Santibáñez-Koref, D. S. Liscia, T. Venesio, J. C. Wang, C. Helms, H. Donis-Keller, D. C. Betticher, H. J. Altermatt, P. R. Hoban, J. Heighway

**Affiliations:** CRC Department of Cancer Genetics, Paterson Institute for Cancer Research, Christie Hospital (NHS) Trust, Manchester, UK.

## Abstract

**Images:**


British Journal of Cancer (1996) 74, 863–870
© 1996 Stockton Press All rights reserved 0007–0920/96 $12.00

### High levels of loss at the 17p telomere suggest the close proximity of a tumour suppressor

GRM White[1], M Stack[2], M Santibáñez-Koref[3], DS Liscia[4], T Venesio[4], J-C Wang[5], C Helms[5], H Donis-Keller[5], DC Betticher[6], HJ Altermatt[6], PR Hoban[1] and J Heighway[1]

*[1]CRC Department of Cancer Genetics, Paterson Institute for Cancer Research, Christie Hospital (NHS) Trust, Wilmslow Road, Manchester M20 4BX, UK; [2]Department of Medicine, Tenovus Building, University of Wales College of Medicine, Cardiff, CF31 4XX, UK; [3]Genome and Gene Evolution Group, MRC Clinical Sciences Centre, Postgraduate Medical School, Hammersmith Hospital, Du Cane Road, London W12 0NN, UK; [4]Servizio di Anatomia Patologica, Ospedale San Giovanni Antica Sede, Dipartimento oncologico USL-1, via Cavour 31, 10123 Turin, Italy; [5]Departments of Genetics, Psychiatry and Surgery, Washington University School of Medicine, St Louis, Missouri 63110, USA and [6]University Hospital of Berne, Berne, Switzerland.*

**Summary** High levels of loss of distal markers on 17p13.3 in breast cancer suggested the presence within the region of at least one tumour-suppressor gene. Here we describe the derivation of two biallelic polymorphisms from the 17p telomeric yeast artificial chromosome (YAC) TYAC98. Polymerase chain reaction–restriction fragment length polymorphism (PCR–RFLP) and multiplex PCR analysis demonstrated that the high level of allelic imbalance observed in breast tumours represented loss of constitutional heterozygosity (LOH) and that this LOH extended to the telomere. Lung carcinoma (but not Wilms' tumour)-derived DNA again revealed a high level of loss of subtelomeric 17p sequences. Telomeric microsatellite polymorphisms from other chromosome arms did not show such elevated loss in either tumour type. This suggested that the 17p loss observed did not reflect a general telomeric instability and provided further evidence for the presence of a breast cancer tumour-suppressor gene in the distal region of 17p13.3.

**Keywords:** chromosome 17; lung cancer; breast cancer; loss of heterozygosity

A highly complex pattern of loss of constitutional heterozygosity (LOH) is observed in human malignancy. Most current models of carcinogenesis assume clonal development of the tumour, with successive genetic lesions conferring an increased proliferative advantage. At the simplest level therefore, every LOH event observed should reflect some such advantage to the malignant cell. However, the situation is likely to be more complicated. If two lesions occur within the same cell at the same time, one conferring a growth advantage the other being silent, then selective pressure will favour the first but both will be present in the resulting clone. This situation may result in confusion over which genomic alterations, in any given tumour, have contributed to the malignant phenotype. It is assumed that if an LOH event occurs at high frequency (>20% of tumours of a particular type) then it is likely to reflect loss of wild-type function of a tumour-suppressor gene encoded within the region of loss.

An individual with a germline mutation in a tumour-suppressor gene might display an increased risk of malignant disease. The individual may pass on this mutation to children who will incur a similar disease risk. Analysis of subsequent tumours often shows that the corresponding normal allele has been deleted in an LOH event. In the case of familial disease, where a genetic linkage or cytogenetic abnormality has helped to target the disease locus, a number of novel tumour-suppressor genes have been identified, including *WT1* (Call *et al.*, 1990), *RB1* (Weinberg, 1990), *BRCA1* (Miki *et al.*, 1994) and *APC* (Joslyn *et al.*, 1991). Conversely, LOH analysis alone has been a historically less successful route to suppressor identification. However, given the imminent localisation of the majority of human genes, the best application of such work might be to provide evidence for the involvement of particular regions in the development of disease. Genes sublocalised to these areas can be quickly examined for a role in malignancy.

Loss of function of the p53 protein, located within 17p13.1, is an important event in the initiation/progression of many human tumours (Baker *et al.*, 1989; Prosser *et al.*, 1990; Mitsudomi *et al.*, 1992). However, LOH studies in breast cancer have suggested the presence of another tumour-suppressor gene mapped to chromosomal region 17p13.3 (Coles *et al.*, 1990; Cornelis *et al.*, 1994; Merlo *et al.*, 1994). Our recent work revealed a complex pattern of LOH in this region in breast tumours (Stack *et al.*, 1995). The highest loss (60–70%), and therefore perhaps the most likely site of the putative suppressor gene, was defined by the three most distal 17p markers, D17S926, D17S695 and D17S849, which were mapped and ordered by fluorescence *in situ* hybridisation (FISH) to a position near the telomere (for physical map of markers, see Stack *et al.*, 1995). Although some tumours showed loss across the whole region studied, many showed retention of more proximal markers, excluding a direct involvement of *TP53*. However, in order to define the extent of the deletions observed and to investigate involvement of the telomere, more distal markers were required.

Yeast artificial chromosomes (YACs), encoding inserts derived from a number of human telomeres, including 17p, were isolated by constructing and screening a single arm, vector library with a TTAGGG 40mer probe (Vocero Akbani *et al.*, 1996). Encoded microsatellite repeat markers were subsequently identified from many telomeres. However, none were isolated from the 17p-encoding YAC. An alternative strategy was therefore employed and the restriction fragment length polymorphism (RFLP) systems derived were used to investigate LOH in several tumour types.

### Materials and methods

### *Patient information and DNA extraction*

Paired primary breast tumours and normal samples were obtained from patients at the S. Giovanni Vecchio Hospital, Turin, Italy as previously outlined (Merlo *et al.*, 1994). Further breast samples and corresponding normals (peripheral blood) were obtained serially from the Withington Hospital, Manchester, UK. Lung samples were obtained from the University Hospital of Berne, Switzerland, from

Correspondence: J Heighway
Received 5 January 1996; revised 17 April 1996; accepted 24 April 1996

patients with resectable staged, non-small-cell lung cancer. The panel included squamous cell carcinomas (L2, L26, L27, L28, L61, L71, L72, L74, L76, L80 and L100), adenocarcinomas (L35, L69, L77 and L91), large-cell carcinoma (L95), carcinoid (L78) and undifferentiated, non-small-cell (L86) samples. In addition to the tumour, normal lung tissue, distant from the tumour site, was resected. Wilms' tumour samples (and peripheral blood controls) were obtained from patients of the Royal Manchester Children's Hospital, Pendlebury, Manchester. Normal DNA for 17TEL allele frequency determinations were derived from samples (blood/normal lung) from the breast/lung cancer series of patients. In all cases DNA was extracted using standard protocols (Sambrook *et al.*, 1989). Approximately 100 ng was used for subsequent polymerase chain reactions (PCRs).

*PCR conditions*

Standard PCR reactions were performed in 25 μl volumes containing a final concentration of 1 × PCR buffer, 100 μM of each dNTP (Promega), 1 μM of each primer and 1 U of *Taq* DNA polymerase. For the *Hha*I polymorphism Dynazyme buffer and polymerase supplied by Flowgen was used. All other PCRs were conducted using buffer and polymerase supplied by either Promega or Boehringer Mannheim. Amplification was performed in an automated thermal cycler (Techne) with a 5 min 97°C denaturation step, then polymerase addition, followed by 35 cycles of 94°C denaturation (96°C for Dynazyme) for 1 min, 55°C (biallelic polymorphisms and vectorette libraries) or 60°C (microsatellites) annealing for 1 min, and extension at 72°C for 1 min. The last cycle was followed by a final extension at 72°C for 10 min.

*Vectorette libraries*

Vectorette libraries (Riley *et al.*, 1990) were constructed using DNA from TYAC98 and digested with several different blunt-end cutting restriction enzymes [*Alu*I, *Hae*III, *Hinc*II, *Rsa*I (all Promega) and *Nla* IV (New England Biolabs)] and ligated to blunt-end vectorette units (Cambridge Research Biochemicals). Restriction digests of 1 μg of TYAC98 DNA were in 50 μl for 2 h at 37°C with 10 U of enzyme (3 U for *Nla*IV) and manufacturers' 1 × buffer. This was followed by enzyme inactivation (15 min at 65°C). Each digest (5 μl) was ligated with T4 DNA ligase (Promega) in 25 μl for 2 h at 37°C with 1.5 pmol of blunt-ended double-stranded vectorette unit and 1 × ligase buffer (Promega). These libraries were then diluted to 250 μl and 1 μl of each library used in PCRs with 1 μM universal vectorette primer (Cambridge Research Biochemicals) and 1 μM of the specific primer under study. Product yield and specificity was improved by dilution of the PCR product 1:100 and reamplification of 1 μl (20 cycles), using the specific primer with a vectorette-nested primer (Cambridge Research Biochemicals).

*Sequencing*

The vectorette-nested primer used to screen the vectorette libraries was biotinylated allowing direct sequencing (Hultman *et al.*, 1989) using Dynabeads M-280 (Dynal). After 90 μl of biotinylated PCR product was bound and strand separated the beads were resuspended in 7 μl of distilled water. This template and 200 ng of specific primer were used in a conventional dideoxy chain termination sequencing reaction (Sequenase v2.0, USB).

*Genotyping and mapping*

A total of 23 CEPH families were found to have parents heterozygous for the *Dde*I RFLP. All available members were genotyped yielding 255 informative meioses. Genotypes were entered into a Macintosh Hypercard application which was used to prepare the input genotype file for the linkage analysis program. Data were transferred to a SPARCstation (Sun Microsystems) for processing. The 17p genotype data were merged with the chromosome 17 marker data in the CEPH database (version 7.1) for the linkage mapping. The computer program CRI-MAP version 2.4 (1989) from P Green (unpublished) was used for two-point and multipoint mapping. The position of 17TEL(*Dde*I) relative to 17p markers was determined (1000:1 odds).

*LOH analysis*

For LOH studies tumour and normal products were run in adjacent gel tracks and the relative allele intensities compared visually. For biallelic 10 μl of PCR product was digested with 10 U restriction enzyme in a 40 μl reaction with 1 × manufacturer's buffer at 37°C for 3 h and the product run on a 2.5% ethidium bromide-stained SEAKEM GTG (FMC) agarose gel. Microsatellite analysis was carried out as previously described (Orphanus *et al.*, 1993). Primer sequences for the telomeric microsatellite repeats studied are shown below:

| | | | |
|---|---|---|---|
| 2p | (sFJS14) | 5′-CTCCACTTAAGCTTGGTTTACA<br>5′-CCCTAAAAGCCTCACTACATG | 119–135 bp<br>(Vocero Akbani *et al.*, 1996) |
| 6q | (sAVA3) | 5′-GGCTAATAAATGCTTAGAGCC<br>5′-GGGCTTAGTTGTTTTCCATTAG | 100–116 bp<br>(Vocero Akbani *et al.*, 1996) |
| 11p | (D11S2071) | 5′-AGGGCAATGAGGACATGAAC<br>5′-ATGTGGCTGGTCCACCTG | 168–202 bp<br>(Browne *et al.*, 1995) |
| 14q | (D14S826) | 5′-TGCTGTTGGACTCAGGTAGCTA<br>5′-TCTCTAAAGCTACTATAACCCAG | 145–161 bp<br>(Pandit *et al.*, 1995) |
| 16q | (D16S303) | 5′-CAACAAGAGCGAAACTCGGTCTCAA<br>5′-GATCAGTGCTCGTTTTTTTTGGTTTGG | 101–115 bp<br>(Shen *et al.*, 1994) |
| 18q | (D18S497)<br>(= D18S70) | 5′-ATTGCCATTCAAGGCTGAAC<br>5′-GTTTTGGGAATGTCAAGAAGTACC | 118–144 bp<br>(Vocero Akbani *et al.*, 1996) |
| 22q | (sJCW16) | 5′-TTGCAGACAGCAGACTACAGG<br>5′-TTCAGTCTGTGGCTGTCCAG | 194–210 bp<br>(Vocero Akbani *et al.*, 1996) |
| Xq | (DXS1108) | 5′-ACTAGGCGACTAATACAGTGGTGC<br>5′-GTGAATTCATCATATGTGATTTCC | 163–177 bp<br>(Frieje *et al.*, 1992) |

*Other microsatellites*

Details of other primer sequences previously reported are: D17S925, D17S926, D17S849 (Gyapay *et al.*, 1994); D17S625 (Utah 269) (Gerken *et al.*, 1995); cCI17.713 (Stack *et al.*, 1994); *TP*53 (Jones and Nakamura, 1992); cCI17-571, D17S1147 (Stack *et al.*, 1995).

*Multiplex PCR*

PCR conditions were as for standard amplifications, with the exception that two primer sets were used in each reaction. The test locus copy number (136 bp product, 17TEL primers/*Dde*I) was compared with the *KRAS2* (196 bp product) locus copy number. Relative intensity of control and test bands were compared visually, with reference to products obtained from DNA isolated from peripheral blood. *KRAS2* exon 1 primers were:

F 5′-GCCTGCTGAAAATGACTGAATATA

R 5′-AATGGTCAGAGAAACCTTTATCTG 196 bp

### Results

*Identification of 17p telomeric polymorphisms*

Analysis of the insert/vector junction of the 150 kb YAC, TYAC98, allowed the identification of a 114 bp STS (sFJS14). This provided the starting point for marker isolation. Vectorette libraries were constructed from digested YAC DNA. Subsequent sequence analysis of a 300 bp

(*Nla*IV library derived) genomic fragment allowed the design of a new insert primer. PCR of normal (peripheral blood) genomic DNA from eight individuals was carried out. To screen for polymorphisms, direct sequencing of the products was carried out. To ensure that even poorly informative markers would be useful, the individuals initially selected were patients of interest. A *Dde*I RFLP, detailed in Table I and Figure 1, was identified.

The sequence was further extended by the design of a new outward facing primer and a further round of PCR with vectorette libraries (*Hae*II library gave a clean band of approximately 300 bp). Sequencing, and subsequent screening of eight individuals with a newly designed reverse primer revealed a second polymorphism (C/T). However, in this case the variation did not alter a restriction site (....TGAG C/T AGGG....). A primer with a near 3′end mismatch (ending 5′....TGcG 3′) was designed. PCR with this primer resulted in the creation of a *Hha*I (GcGC or GcGT) RFLP (Table I, Figure 2). Amplification with *Taq* DNA polymerase (Promega) produced surprising results. Subsequent digestion of heterozygote samples with *Hha*I suggested that the PCR had favoured one (cutting) allele in all cases. It was assumed that this was due to unequal PCR amplification efficiency as a result of differing degrees of secondary structure, dependent on the particular base present at the polymorphic site (primer-binding site adjacent to this base). Using Dynazyme

**Table I** Details of the two biallelic polymorphisms that combine to give 17TEL

| *RFLP* | *Primers* | *Sequence* | *Allele* | *Allele size (bp)* | *Frequency For* Dde *I n=48 For* Hha *I n=69* | *Heterozygosity (%) Expected* | *Observed* |
|---|---|---|---|---|---|---|---|
| *Dde* I | 94-649 | TCC AGC TAG AAC TTC GTG CA | $a_1$ | 136 | 0.23 | 35.4 | 37.5 |
| | 95-450 | TTC TCC CTT GTT TGG TTG GA | $a_2$ | 92 (+44) | 0.77 | | |
| *Hha* I | 95-448 | CCT GTG GGT TTG TCA GCA GT | $a_3$ | 145 | 0.14 | 24.1 | 21.7 |
| | 95-463 | TAA GGT GTC TCG AGG TGc G | $a_4$ | 126 (+19) | 0.86 | | |

Primer 95-463 has a mismatch one base from the 3′ end (lower case).

**Table II** Loss of heterozygosity study in 39 breast cancer patients

| *Sample* | *cCI17-713* | *YNZ22* | *Utah 20* | *YNH37.3* | *cCI17-571* | *D17S1174* | *D17S926* | *D17S695* | *D17S849* | *17TEL* |
|---|---|---|---|---|---|---|---|---|---|---|
| 573 | ○ | ○ | – | – | – | – | ● | ○ | ○ | – |
| 584 | ○ | – | – | – | – | – | – | ◐ | ● | ○ |
| 636 | ○ | ○ | ● | – | – | – | ○ | ○ | ○ | – |
| 654 | | ○ | ○ | – | ○ | – | – | ● | – | – |
| 658 | ○ | ○ | | – | ◐ | – | ● | ● | ● | – |
| 661 | ● | ● | ◐ | | ○ | – | ● | ● | ● | ● |
| 683 | ○ | ○ | – | ● | – | – | ○ | ◐ | ● | ● |
| 709 | ○ | – | ○ | – | – | – | – | ◐ | ● | – |
| 720 | – | ○ | ● | ○ | ● | – | – | – | ○ | – |
| 731 | ○ | ○ | ○ | – | ○ | – | – | ● | – | ○ |
| 786 | | ○ | ● | ◐ | ◐ | – | – | ○ | ○ | – |
| 788 | | – | ○ | – | ○ | – | | ● | – | ● |
| 792 | ○ | ● | ○ | – | ◐ | ◐ | | ● | ○ | – |
| 682 | ● | ● | – | ◐ | ○ | – | – | ● | ● | ● |
| 594 | ● | ● | | ○ | ○ | – | – | ● | ● | – |
| 518 | ○ | ○ | ○ | ● | ○ | – | ○ | ○ | – | ○ |
| 486 | ○ | ○ | ● | ◐ | ● | ● | ◐ | ● | ● | ● |
| 446 | ● | ● | – | – | ◐ | ● | ● | ● | ● | – |
| 514 | | ● | ● | ● | – | – | ● | ● | ● | ○ |
| 515 | ● | ● | ● | | | | | ● | – | – |
| 548 | ● | – | ● | – | ● | – | ● | ● | – | ● |
| 572 | ● | ● | ● | – | – | – | – | ● | ● | – |
| 578 | | ● | – | – | ● | – | – | – | – | ● |
| 591 | – | ● | | – | – | – | ● | ◐ | ● | ● |
| 670 | ● | ● | – | – | ● | ● | – | – | ● | – |
| 721 | – | ● | ● | – | ● | – | – | ◐ | ○ | – |
| 722 | ● | ● | ○ | ● | ● | – | | ● | – | ● |
| 729 | ◐ | ○ | ○ | – | ◐ | ◐ | ◐ | ◐ | – | – |
| 492 | ○ | ○ | | | ○ | ○ | ○ | ○ | – | – |
| 551 | ○ | ○ | ○ | – | ○ | – | – | ◐ | ○ | – |
| 581 | ○ | ○ | ○ | ○ | ○ | – | – | ○ | ○ | – |
| 593 | – | ○ | ○ | – | ◐ | – | – | ○ | ○ | ○ |
| 637 | – | ○ | ○ | ○ | ○ | – | – | ○ | | – |
| 704 | ○ | ○ | ○ | – | ○ | – | – | – | ○ | ○ |
| 692 | ○ | ○ | ◐ | ○ | – | – | – | ○ | | ● |
| 700 | ○ | – | – | – | – | – | – | – | ○ | – |
| 730 | ○ | ○ | – | – | | ○ | – | ○ | – | ○ |
| 733 | ◐ | ○ | – | ○ | | – | | ○ | – | – |
| 782 | ○ | ○ | – | – | ○ | – | – | ◐ | – | – |
| Loss/total | 11/29 | 13/34 | 11/24 | 7/13 | 13/26 | 5/7 | 9/13 | 23/34 | 13/24 | 10/17 |
| Loss (%) | 38 | 38 | 46 | 54 | 50 | 71 | 69 | 68 | 54 | 59 |

This table was previously reported (Stack *et al*., 1995) with the exception of the 17TEL results. ●, clear loss; ◐, marginal imbalance; ○, no loss; –, non-informative samples. Samples where no information was obtained are left blank. Values for percentage loss of each marker are given at the foot of the table.

(Flowgen) in place of *Taq* DNA polymerase overcame these effects. These two linked polymorphisms are subsequently referred to as 17TEL.

*Confirmation of subtelomeric location of 17TEL*

Fluorescent *in situ* hybridisation (FISH) analysis placed the human sequence carried by TYAC98 distal to all 17p13.3 YACs tested that encoded known genes or Genethon markers (Stack *et al.*, 1995). However, there remained a small possibility that the YAC insert carried a large internal deletion, and that the 17TEL RFLP markers were derived from a small proximal component (not seen by FISH). Multipoint linkage analysis, using the *Dde*I RFLP and Genethon markers, placed 17TEL 4.1 cM (sex average, male 7.2 cM, female 1.3 cM, 1000 : 1 odds) telomeric from the two most distal Genethon markers (D17S926 and D17S849) used in the initial study (Stack *et al.*, 1995). The results suggested that the RFLP system lay within 150 kb of the 17p telomere. 17p linkage information, including genotypes, will be available through the WWW Genlink Resource (http://www.genlink.wustl.edu/).

*17TEL LOCH results*

Table II details our previous LOH study on 17p and is reproduced here with the additional 17TEL data. Of the 17 samples heterozygous for 17TEL, 10 (59%) were found to show loss. In the four cases where the samples were heterozygous for both polymorphisms, they gave identical findings. There was a tendency for the telomeric polymorphism to be lost if distal markers were also lost (nine cases, e.g. 661), and retained if distal markers were retained (four cases, e.g. 518). But there were also three cases where the telomere was not lost when distal markers were (e.g. 584) and one case (692) where the telomere was lost and distal markers were not.

A similar LOH study was conducted using resectable staged, non-small-cell lung tumours, the results of which are shown in Table III. Here the pattern of loss is less complex than in the breast tumours, with 13 out of 18 (72%) showing loss of all informative markers on the p arm including *TP53* (e.g. L61) and four out of 18 (22%) showing no loss of any informative markers (e.g. L26); this includes case L71 which has marginal imbalance for 17TEL only. This level of loss is higher than other reports (Chiba *et al.*, 1990; Hiyama *et al.*, 1995) perhaps because of differences in tumour source or sample preparation. Sample L74 shows loss of distal markers but this does not apparently include *TP53*. The level of loss for the more proximal marker D17S925 (one out of 13 informative samples) would not be considered significant and is what might be expected from a region unlinked to tumorigenesis. Interestingly, marginal imbalance, often seen with the breast tumours, was much rarer with the lung samples. A smaller study was also conducted using Wilms' tumour and the three distal markers D17S926, D17S695, and D17S849. Out of 17 paired samples, only two showed loss and only for one marker (results not shown).

Throughout, we have used the terminology of LOH. But as this is a PCR-based study, it would be more correct to use the term allelic imbalance. However, we have some evidence that the observed imbalances are due to true loss and not amplification events. A total of 18 DNA samples showing

**Figure 1** Ethidium bromide-stained gel showing the *Dde*I polymorphism. Tumour DNA is represented as T and normal DNA (corresponding peripheral blood) is represented as N. The more common (restricted) allele is 92bp with the uncut 136 bp, the 44 bp fragment is barely visible. Sample 1 is homozygous cutting and demonstrates full enzyme digestion. Sample 4 is homozygous non-cutting, and samples 2 and 3 demonstrate two examples of LOH in breast cancer (cases 548 and 682 respectively).

**Figure 2** Ethidium bromide-stained gel showing the *Hha*I polymorphism. Tumour DNA is represented as T and normal DNA (corresponding peripheral blood) is represented as N. The more common (restricted) allele is 126 bp with the uncut 145 bp, the 19 bp fragment is not visible. Sample 5 is homozygous cutting and demonstrates full enzyme digestion. Sample 7 is homozygous non-cutting while sample 6 is an example of LOH in breast cancer (case 88/344).

**Table III** Loss of heterozygosity study in 18 resectable, staged, non-small-cell lung cancer patients

| *Sample* | *D17S925* | TP53 | *cCI17-713* | *cCI17-571* | *D17S1174* | *D17S926* | *D17S695* | *D17S849* | *17TEL* |
|---|---|---|---|---|---|---|---|---|---|
| L2 | ○ | – | ● | ● | ● | ● | – | ● | – |
| L26 | – | ○ | – | ○ | – | ○ | ○ | – | ○ |
| L27 | ○ | – | ● | ● | ● | ● | ● | ● | – |
| L28 | – | – | – | ● | ● | ● | – | ● | ● |
| L35 | ○ | – | ○ | ○ | ○ | ○ | ○ | ○ | – |
| L61 | ○ | ● | – | – | ● | ● | ● | – | ● |
| L69 | – | ● | – | – | – | ● | ● | ● | ● |
| L71 | ○ | ○ | ○ | – | ○ | – | ○ | – | ◍ |
| L72 | ● | – | ● | – | ● | ● | ● | ● | – |
| L74 | ○ | ○ | ○ | – | ● | ● | ● | ● | – |
| L76 | | – | ● | – | – | ● | ● | ● | – |
| L77 | ○ | ● | ● | ● | ● | ● | ● | – | – |
| L78 | | – | ○ | – | ○ | ○ | ○ | – | – |
| L80 | ○ | ● | ● | ● | ● | ● | ● | ● | ● |
| L86 | ○ | ● | – | ● | ● | ● | ● | ● | – |
| L91 | ○ | ● | ● | ● | ● | ● | ● | ● | ● |
| L95 | ○ | – | ● | ● | ● | ● | – | ● | – |
| L100 | ○ | ● | ● | ● | – | ● | ● | ● | – |

All markers map to 17p13 except D17S925 which is located proximal. ●, clear loss; ◍, marginal imbalance; ○, no loss; –, non-informative samples. Samples where no information was obtained are left blank. Values for percentage loss for each marker are given at the foot of the table.

LOH of 17TEL (lung and breast cancer) were used in multiplex PCR assays. Although multiplex PCR is not sufficiently sensitive to measure small differences in starting target ratios (such as those that might be generated by an LOH event), large target ratio differences (such as those created by gene amplification) may be detected with relative ease (Betticher *et al.*, 1996). Two sets of primers (*Dde*I RFLP, 136 bp and *KRAS2*, 196 bp) were incorporated in each reaction. Following PCR, relative band intensity provided a measure of the relative starting ratios of each locus in the tumour sample. Gene amplification would result in strengthening of the intensity of the band derived from the amplified gene. In comparison with results from normal control DNA, no evidence for amplification of 17TEL was obtained in any tumour sample, suggesting that the allelic imbalance seen at the telomere reflected true LOH events. Similarly, to exclude homozygous deletion of the marker, all breast and lung tumours demonstrating apparent retention of 17TEL (PCR product may derive from a residual and minority, normal cell component of the tumour) were analysed by multiplex PCR (Figure 3). In no case did the data suggest that homozygous deletion of the region had occurred.

**Figure 3** Multiplex PCR analysis of tumour and normal DNA samples. Tumour samples (T, case as numbered) were compared with corresponding normal DNA (N) from the patients. Tumour samples 682 and 548 demonstrated allelic imbalance in 17TEL markers. The multiplex analysis indicated that these imbalances were not the result of amplification events. Tumour samples 731 and 584 showed no allelic imbalance for 17TEL. The multiplex suggested that homozygous loss of the 17TEL marker region (analysed PCR product derived from normal cell component of tumour) had not occurred. End track is $\phi$X174/*Hae*III marker.

### *Telomeric imbalance on other chromosomes*

To demonstrate that the high level of loss observed at 17p13.3 in breast cancer was not a general property of telomere-associated sequences in tumours, microsatellite repeat polymorphisms from other chromosome arms were studied. As a result of the relatively low percentage heterozygosity of the biallelic polymorphisms, 17TEL data was available only on a restricted number of cases. It was decided to increase the number of breast tumours in the study. Tumours were subsequently divided into two groups, those showing LOH for 17TEL (17L) and those not showing LOH (17NL). Table IV shows the telomeric marker and loss pattern for those breast cancer samples showing LOH for 17TEL. Table V shows the loss pattern for those not showing LOH for 17TEL. With the small numbers involved, significant differences were unlikely, but it is clear that no other telomeric region analysed showed loss at levels higher than 17p. Indeed, in general, LOH levels were much lower. There seems to be no great difference between the 17L and 17NL groups suggesting the absence of a subset of tumours that shed subtelomeric sequences. However, the 17NL group does appear to show a slightly lower overall level of LOH. This could perhaps be explained by the unintentional selection of some tumours with a high normal component (10 out of 21 samples showed no loss at any of the markers).

The lung samples were also studied for LOH with the telomeric markers. The results, as shown in Table VI; were similar to those obtained for the breast samples with a low level of loss recorded on other autosomes.

### Discussion

We have previously reported evidence for the existence of a breast cancer-suppressor gene located within the distal region of 17p13.3. This study, reporting the identification of a novel four-allele RFLP system mapped to the 17p subtelomeric region, extends those initial observations. In almost all cases examined, the loss region incorporating the distal markers extends to the telomere. Could we therefore be justified in ascribing the LOH events observed to simple loss of subtelomeric sequences in an unstable genome? This explanation would seem unlikely, given that the levels of loss at the distal regions of other chromosomes occurred at a substantially lower frequency. We would therefore argue that the breast tumour data presented in this study support the hypothesis that a p53-independent suppressor gene resides within the subtelomeric region of 17p13.3.

Although both breast and lung cancers are epithelial in origin and have known p53 involvement (Prosser *et al.*, 1990; Mitsudomi *et al.*, 1992), there is a marked difference between

**Table IV** Results of LOH study using eight microsatellite markers in 16 breast cancer patients that show 17TEL loss

| *Sample* | *2p* | *6q* | *11p* | *14q* | *16q* | *18q* | *22q* | *Xq* |
|---|---|---|---|---|---|---|---|---|
| 486 | – | | – | | – | ○ | – | – |
| 548 | ● | ○ | ● | ● | – | ● | ● | – |
| 578 | ○ | | ○ | ● | – | ○ | | ● |
| 591 | ● | – | ● | ● | – | | – | ○ |
| 661 | ○ | ○ | ○ | ● | ○ | ● | ● | ○ |
| 682 | – | | – | ○ | – | – | ○ | ○ |
| 683 | ○ | – | ○ | ● | – | ○ | – | ● |
| 692 | ○ | ○ | ○ | ○ | – | ● | ○ | ○ |
| 722 | – | – | ● | – | – | – | | – |
| 788 | ○ | | ○ | – | – | | ○ | – |
| 88-153 | ○ | – | ○ | ○ | ○ | ○ | ○ | – |
| 88-228 | ○ | – | ○ | – | ○ | ○ | ○ | ○ |
| 88-343 | ○ | ● | ● | ○ | – | ○ | ○ | ○ |
| 88-389 | | ● | | ○ | – | ● | – | ○ |
| 88-397 | ○ | ○ | – | | – | ○ | – | ○ |
| 89-31 | ○ | – | | ○ | ● | | ○ | ○ |
| 89-51 | ○ | ○ | – | – | – | | – | ○ |
| Loss/total | 2/13 | 2/7 | 4/11 | 5/11 | 1/4 | 4/11 | 2/9 | 2/12 |
| Loss (%) | 15 | 29 | 36 | 45 | 25 | 36 | 22 | 17 |

●, loss; ○, no loss; –, non-informative samples. Samples where no information was obtained are left blank. Values for percentage loss for each marker are given at the foot of the table.

**Table V** Results of LOH study using eight microsatellite markers in 21 breast cancer patients that show no 17TEL loss

| *Sample* | *2p* | *6q* | *11p* | *14q* | *16q* | *18q* | *22q* | *Xq* |
|---|---|---|---|---|---|---|---|---|
| 514 | ○ | ○ | ○ | ○ | – | – | ● | ○ |
| 518 | ○ | ○ | ○ | ○ | ● | ○ | – | ○ |
| 535 | – | – | ● | – | – | – | – | – |
| 584 | – | ○ | ○ | ● | – | ○ | | – |
| 594 | – | ● | ● | – | – | ○ | – | – |
| 704 | ○ | ● | ○ | ● | ● | ● | ● | ○ |
| 730 | ○ | ● | ○ | ○ | – | ○ | ○ | ● |
| 731 | ○ | ○ | ○ | ○ | ● | ○ | ○ | ○ |
| 640 | ○ | ○ | ○ | – | – | ○ | ○ | ○ |
| 641 | ○ | – | ○ | ○ | ○ | | ○ | ○ |
| 88-139 | ○ | ○ | ○ | ○ | – | ○ | – | ○ |
| 88-226 | ○ | ○ | ○ | ○ | ● | ● | ○ | – |
| 88-361 | ○ | ○ | – | ○ | – | ○ | ○ | ○ |
| 88-19 | ○ | ○ | ○ | ○ | ○ | ○ | ○ | ○ |
| 89-22 | ○ | – | – | ○ | – | ○ | ○ | |
| 89-76 | ○ | ○ | ○ | ○ | ○ | ○ | ○ | ○ |
| 89-86 | ○ | – | | ○ | ○ | ○ | ○ | – |
| 89-129 | ○ | ○ | ○ | ○ | ● | ○ | ○ | ● |
| 89-139 | ○ | ○ | ○ | – | – | ○ | ○ | ○ |
| 89-163 | ○ | – | ○ | ○ | – | ○ | | ○ |
| 89-372 | ○ | ● | ○ | ○ | ○ | ○ | ○ | – |
| Loss/total | 0/18 | 4/16 | 2/18 | 2/17 | 5/10 | 2/18 | 2/15 | 2/14 |
| Loss (%) | 0 | 25 | 11 | 12 | 50 | 11 | 13 | 14 |

●, loss; ○, no loss; –, non-informative samples. Samples where no information was obtained are left blank. Values for percentage loss for each marker are given at the foot of the table.

their 17p LOH patterns. Different again were the results for Wilms' tumour, a paediatric embryonal tumour of the kidney, in which only very low levels of loss were seen. Lung tumours generally showed either complete loss or, alternatively, no loss for all markers on the 17p arm. This LOH for large regions of chromosome 17 in lung tumours perhaps shows some similarity to the pattern described in sporadic ovarian carcinomas, where loss of the entire chromosome is a very common event (Foulkes *et al.*, 1993). The LOH pattern for breast cancers was much more complex, with individual samples showing both loss and no loss for different markers along the 17p arm. It is interesting to speculate on the observation that the region of loss on 17p for lung tumours is so large, possibly including the entire 17p arm. It is possible that *TP53* (or another suppressor) is the sole target of the LOH events. In this case the large region of loss could be a result of the particular carcinogenic agents that lung tissue is exposed to. Alternatively, defects within two or more separate and distant genes situated on 17p may be required for tumorigenesis. Possible support for the existence of multiple 17p suppressor targets is provided by the observation of LOH at the *TP53* locus in lung tumours in the absence of a detectable mutation in the remaining allele (Hiyama *et al.*, 1995).

To our knowledge, this is the first major study of the loss of telomere-associated sequences in malignant disease. The levels of loss found, in both lung and breast cancer, for the majority of telomeric markers was, in the main, similar to the levels considered to be around the borderline for significant involvement of a region in the development of a tumour type (10–20%). We are therefore unable to support the idea that subtelomeric sequences are highly unstable in malignant disease. Given that telomeres are thought to be required for chromosome stability, the high level of loss of 17TEL observed might appear surprising. However, if the loss mechanism was primarily one of somatic recombination and reduplication following the LOH event, both 17 homologues would have functional telomeres. Alternatively, if the LOH events derived from breakage and loss of the chromosome end, it is possible that telomerase, activated in the malignant cells, might be involved in re-creation of a functional telomere at the break site. Telomere capture (Meltzer *et al.*, 1993) is a further mechanism by which a chromosome might reacquire a functional terminus.

**Table VI** Results of LOH study using six microsatellite markers in 18 lung cancer patients

| *Sample* | *2p* | *6q* | *14q* | *16q* | *18q* | *22q* |
|---|---|---|---|---|---|---|
| L2 | ● | – | ○ | – | ○ | – |
| L26 | ○ | ○ | ○ | – | – | ○ |
| L27 | – | ● | ○ | – | ● | ○ |
| L28 | ○ | ○ | – | – | ○ | ○ |
| L35 | ○ | ○ | – | ○ | ○ | ○ |
| L61 | ○ | ○ | ● | ● | ● | ● |
| L69 | – | ● | – | – | ○ | ○ |
| L71 | ● | – | ○ | ○ | ○ | ○ |
| L72 | – | ○ | – | ● | – | ● |
| L74 | – | ○ | ○ | ● | ○ | ● |
| L76 | ○ | – | ○ | – | ● | ○ |
| L77 | ○ | ○ | – | – | – | ● |
| L78 | ○ | – | ○ | ● | ○ | – |
| L80 | ○ | ○ | ● | ○ | ○ | ○ |
| L86 | ○ | ○ | ○ | – | ○ | ○ |
| L91 | ● | ○ | – | – | ○ | ● |
| L95 | ○ | ○ | – | ○ | ● | – |
| L100 | ○ | ○ | | ○ | ○ | – |
| Loss/total | 3/14 | 2/14 | 2/10 | 4/9 | 4/15 | 5/14 |
| Loss (%) | 21 | 14 | 20 | 44 | 27 | 36 |

●, loss; ○, no loss; –, non-informative samples. Samples where no information was obtained are left blank. Values for percentage loss for each marker are given at the foot of the table.

The primary aim of this work was to provide evidence for the existence of and fine location of, a 17p13.3-encoded suppressor gene. Although the region showed very high levels of loss in lung cancer, strongly supporting the existence of a 17p tumour suppressor, the homogeneity of the deletions and size of the region involved meant that almost no fine positional information was derived. In contrast, while still showing high LOH levels for distal markers, many breast tumours showed a more heterogeneous 17p13.3 loss pattern, from which it might be possible to derive positional information. However, we are faced with a problem. If we assume that a single tumour suppressor resides within a region (as might be the case with an inherited predisposition) then mapping of a minimal region of deletion (MRD) might indicate the position of that gene. But if more than one tumour suppressor is present within a region (providing multiple, potential LOH targets), MRD mapping may lead to an investigation of the wrong area of the genome. It might be possible to overcome this limitation by using very large numbers of tumours but generally, in the absence of a familial syndrome and linkage data, we are unable to decide whether one or multiple targets exist. In addition, a further layer of complexity is present in PCR-based studies, given the possibility that areas of homozygous deletion can appear as regions of retained heterozygosity, through amplification of the normal cell component of the tumour sample (Williamson *et al*., 1995). However, we find no evidence for homozygous deletion at the 17p telomere. Given these limitations and assuming a single target gene model (which we have no evidence for) our data would suggest that a breast cancer tumour suppressor lies between D17S926 and the telomere (cases 658, 661, 683 and 731). Future assignment of genes and expressed sequence-tagged sites to this region will allow us to test this hypothesis.

**Acknowledgements**
We are grateful to Nigel Barron for photographic and artwork. We would also like to thank Dr Anna Kelsey, Royal Manchester Children's Hospital for provision of the Wilms' tumours. This research was funded in part by the Cancer Research Campaign, UK, in part by NIH grant (HG00100) to H Donis-Keller and in part by the Associazione Italiana Ricerca Cancro.

### References

BAKER SJ, FEARON ER, NIGRO JM, HAMILTON SR, PREISINGER AC, JESSUP JM, VAN TUINEN P, LEDBETTER DH, BARKER DF, NAKAMURA Y, WHITE R AND VOGELSTEIN B. (1989). Chromosome 17 deletions and p53 gene mutations in colorectal carcinomas. *Science*, **244**, 217–221.

BETTICHER DC, HEIGHWAY J, HASELTON PS, ALTERMATT HJ, RYDER WDJ, CERNY T AND THATCHER N. (1996). Prognostic significance of *CCND1* (cyclin D1) overexpression in lung cancer. *Br. J. Cancer*, **73**, 294–300.

BROWNE, DL, SMITH BA, DIETZ-BAND J, RIETHMANN HC, PHROMCHOTIKUL T AND LITT M. (1995). Dinucleotide repeat polymorphism at the human chromosome 11p telomere (D11S2071) *Genomics*, **25**, 600–601.

CALL KM, GLASER T, ITO CY, BUCKLER AJ, PELLETIER J, HABER DA, ROSE EA, KRAL A, YEGER H, LEWIS WH, JONES C AND HOUSMAN DE. (1990). Isolation and characterization of a zinc finger polypeptide gene at the human chromosome 11 Wilms' tumour locus. *Cell*, **60**, 509–520.

CHIBA I, TAKAHASHI T, NAU MM, D'AMICO D, CURIEL DT, MITSUDOMI T, BUCHHAGEN DL, CARBONE D, PIANTADOSI S, KOGA H, REISSMAN PT, SLAMON DJ, HOLMES EC AND MINNA JD. (1990). Mutations in the p53 gene are frequent in primary, resected non-small cell lung cancer. *Oncogene*, **5**, 1603–1610.

COLES C, THOMPSON AM, ELDER PA, COHEN BB, MACKENZIE IM, CRANSTON G, CHETTY U, MACKAY J, MACDONALD M, NAKAMURA Y, HOYHEIM B AND STEEL CM. (1990). Evidence implicating at least two genes on chromosome 17p in breast carcinogenesis. *Lancet*, **336**, 761.

CORNELIS RS, VAN VLIET M, VOS CBJ, CLETON-JANSEN A-M, VAN DE VIJVER MJ, PETERSE JL, KHAM M, BØRRESEN A-L, CORNELISSE CJ AND DEVILEE P. (1994). Evidence for a gene on 17p13.3, distal to *TP53* as a target for allele loss in breast tumours without p53 mutations. *Cancer Res.*, **54**, 4200–4206.

FOULKES WD, BLACK DM, STAMP GHM, SOLOMON E AND TROWSDALE J. (1993). Very frequent loss of heterozygosity throughout chromosome 17 in sporadic ovarian carcinoma. *Int. J. Cancer*, **54**, 220–225.

FRIEJE D, HELMS C, WATSON MS AND DONIS-KELLER H. (1992). Identification of a second autosomal region near the Xq and Yq telomeres. *Science*, **258**, 1784–1787.

GERKEN SC, ALBERTSEN H, ALSNER T, BALLARD L, HOLIK P, LAWRENCE E, MOORE M, ZHAO X AND WHITE R. (1995). A strategy for constructing high-resolution genetic maps of the human genome: a genetic map of chromosome 17p, ordered with meiotic breakpoint-mapping panels. *Am. J. Hum. Genet.*, **56**, 484–499.

GYAPAY G, MORISSETTE J, VIGNAL A, DIB C, KIZAMES C, MILLASSEAU P, MARC S, BERNADI G, LATHROP M AND WEISSENBACH J. (1994). The 1993–94 Généthon human genetic linkage map. *Nature Genet.*, **7**, 246–339.

HIYAMA K, ISHIOKA S, SHIROTANI Y, INAI K, HIYAMA E, MURAKAMI I, ISOBE T, INAMIZU T AND YAMAKIDO M. (1995). Alterations in telomeric repeat length in lung cancer are associated with loss of heterozygosity in p53 and Rb. *Oncogene*, **10**, 937–944.

HULTMAN T, STÅHL S, HORNES E AND UHLÉN M. (1989). Direct solid phase sequencing of genomic and plasmid DNA using magnetic beads as solid support. *Nucleic Acids Res.*, **17**, 4937–4946.

JONES MH AND NAKAMURA Y. (1992). Detection of loss of heterozygosity at the human *TP53* locus using a dinucleotide repeat polymorphism. *Genes Chrom. Cancer*, **5**, 89–90.

JOSLYN J, CARLSON M, THLIVERIS A, ALBERTSEN H, GELBERT L, SAMOWITZ W, GRODEN J, STEVENS J, SPIRIO L, ROBERTSON M, SARGEANT L, CRAPCHO K, WOLFF E, BURT R, HUGHES JP, WARRINGTON J, WASMUTH J, LE PASLIEF D, ABDERRAHIM H, COHEN D, LEPPERT M AND WHITE R. (1991). Identification of deletion mutants and three new genes at the familial polyposis locus. *Cell*, **66**, 601–613.

MELTZER PS, GVAN XY AND TRENT JM. (1993). Telomere capture stabilizes chromosome breakage. *Nature Genet.*, **4**, 252–255.

MERLO GR, VENESIO T, BERNARDI A, CROPP CS, DIELLA F, CAPPA APM, LISCIA DS AND CALLAHAN R. (1994). Evidence for a second tumour suppressor gene on 17p linked to high S-phase index in primary human breast carcinomas. *Cancer Genet. Cytogenet.*, **76**, 106–111.

MIKI Y, SWENSEN J, SHATTUCK-EIDENS D, FUTREAL PA, HARSHMAN K, TAVTIGIAN S, LIU Q, COCHRAN C, BENNET LM, DING W, BELL R, ROSENTHAL J, HUSSEY C, TRAN T, MCCLURE M, FRYE C, HATTIER T, PHELPS R, HAUGEN-STRANO A, KATCHER H, YAKUMO K, GHOLAMI Z, SHAFFER D, STONE S, BAYER S, WRAY C, BOGDEN R, DAYANANTH P, WARD J, TONIN P, NAROD S, BRISTOW PK, NORRIS FH, HELVERING L, MORRISON P, ROSTECK P, LAI M, BARRET JC, LEWIS C, NEUHAUSEN S, CANNON-ALBRIGHT L, GOLDGAR D, WISEMAN R, KAMB A AND SKOLNICK MH. (1994). A strong candidate gene for the breast and ovarian cancer susceptibility gene. *Science*, **266**, 66–71.

MITSUDOMI T, STEINBERG SM, NAU MM, CARBONE D, D'AMICO D, BODMER S, OIE HK, LINNOILA RI, MULSHINE JL, MINNA JD AND GAZDAR AF. (1992). P53 gene mutations in non-small-cell lung cancer cell lines and their correlation with the presence of *ras* mutations and clinical features. *Oncogene*, **7**, 171–180.

ORPHANUS V, MCGOWN G, BOYLE JM AND SANTIBÁNEZ-KOREF M. (1993). Thirteen dinucleotide repeat polymorphisms on human chromosome 6. *Hum. Mol. Genet.*, **2**, 2196.

PANDIT SD, WANG JC, VEILE RA, MISHRA SK, WARLICK CA AND DONIS-KELLER H. (1995). Index, comprehensive microsatellite and unified linkage maps of human chromosome 14 with cytogenetic tie points and a telomere microsatellite marker. *Genomics*, **29**, 653–664.

PROSSER J, THOMPSON AM, GRANSTON G AND EVANS HJ. (1990). Evidence that p53 behaves as a tumour suppressor gene in sporadic breast tumours. *Oncogene*, **5**, 1573–1579.

RILEY JH, BUTLER R, OGILVIE D, FINNIEAR R, JENNER D, POWELL S ANAND R, SMITH JC. AND MARKHAM AF. (1990). A novel, rapid method for the isolation of terminal sequences from yeast artificial chromosomes (YAC) clones. *Nucleic Acids Res.*, **18**, 2887–2890.

SAMBROOK J, FRITSCH EF AND MANIATIS T. (1989). *Molecular Cloning: A Laboratory Manual* 2nd edition. Cold Spring Harbor Laboratory Press: Cold Spring Harbor, NY, USA.

SHEN Y, KOZMAN HM, THOMPSON A, PHILIPS HA, HOLMAN K, NANCARROW J, LANE S, CHEN LZ, APOSTOLOU S, DOGGET NA, CALLEN DF, MULLEY JC, SUTHERLAND GR AND RICHARDS RI. (1994). A PCR based linkage map of human chromosome 16. *Genomics*, **22**, 68–76.

STACK M, CRICHTON D AND SANTIBANEZ-KOREF MF. (1994). Two dinucleotide repeat polymorphisms at 17p13. *Hum. Mol. Genet.*, **3**, 1443.

STACK M, JONES D, WHITE G, LISCIA DS, VENESIO T, CASEY G, CRICHTON D, VARLEY J, MITCHELL E, HEIGHWAY J AND SANTIBÁNEZ-KOREF M. (1995). Detailed mapping and loss of heterozygosity analysis suggests a suppressor locus involved in sporadic breast cancer within a distal region of chromosome band 17p13.3. *Hum. Mol. Genet.*, **4**, 2047–2055.

VOCERO AKBANI A, HELMS C, WANG JC, SANJURJO FJ, KORTE-SARFATY J, VEILE RA, LIU L, JAUCH A, CREMER T, BURGESS AK, HING AV, HOLT MS, RAMACHANDRA S, WHELAN AJ, ANKER R, AHRENT L, CHEN M, GAVIN MR, IANNANTUONI K, MORTON SM, PANDIT SD, READ CM, STEINBRUECK T, WARLICK C, SMOLLER DA AND DONIS-KELLER H. (1996). Mapping human telomere regions with YAC and P1 clones: chromosome specific markers for 27 telomeres including 147 STSs and 24 polymorphisms for 14 proterminal regions. *Genomics*, (submitted).

WEINBERG RA. (1990). The retinoblastoma gene and cell growth control. *Trends Biochem. Sci.*, **15**, 199–202.

WILLIAMSON MP, ELDER PA, SHAW ME, DEVLIN J AND KNOWLES MA. (1995). *p16(CDKN2)* is a major deletion target at 9p21 in bladder cancer. *Hum. Mol. Genet.*, **4**, 1569–1577.